# Sustainable Engineering Design in Education: A Pilot Study of Teaching Right‐to‐Repair Principles through Project‐Based Learning

**DOI:** 10.1002/gch2.202300158

**Published:** 2023-10-03

**Authors:** Sam Fishlock, Matthew Thompson, Anoop Grewal

**Affiliations:** ^1^ The Engineering and Design Institute TEDI‐London Building 11 Quebec Way London SE16 7LG UK; ^2^ Arizona State University GWC 416 Tempe AZ 85287 USA

**Keywords:** project‐based learning, right‐to‐repair, UN sustainable development goals

## Abstract

Over 60 million tons of E‐waste is expected to be generated in 2023, with associated significant impacts on health and the environment. To reduce the number of products sent to landfills, “Right to Repair” (RtR) movements are gaining momentum in many countries, including the UK, USA, and EU member states. While Universities are seen as important stakeholders to drive forward sustainable design practices, there is currently little work looking at training undergraduate design engineers in the principles of designing household products in support of RtR. In particular, the project‐based learning (PBL) pedagogy shows promise in engaging and training students with the skills and knowledge required to successfully design products for RtR. In this paper, a pilot‐study of teaching engineers is presented to design products compatible with RtR principles, alongside many technical skills, in a first‐year PBL course. The key outputs of this paper are the design of the module, which can be used to help inform first‐year engineering education, the high engagement of students, with 100% of respondents agreeing that they intend to try to implement sustainable design practices in future, and some of the innovative features that students implement in their projects.

## Graphical abstract

The right to repair movement is growing and aims to ensure that all the products are repaired, so that they last longer, which is a key way to reduce E‐Waste. Here, the design and implementation of a project‐based learning university course is presented to help embed the tools for designing for the right to repair.

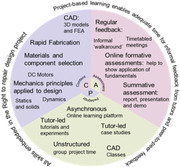

## 1 Introduction and Literature Survey

### 1.1 The Right to Repair

The right‐to‐repair (RtR) movement aims to ensure that consumers have the right to simply and cost‐effectively repair and fix their products and equipment, including electronic and consumer goods.^[^ ^1^ ^]^ Implementing the RtR is seen as a way to improve consumer rights, for example, Hanley et al. stated that “when consumers cannot repair, modify, or tinker with the goods they have rightfully purchased, consumers do not fully own their products”.^[^ ^2^ ^]^ Alongside greater consumer rights, RtR can help to reduce E‐Waste, the generation of which is expected to reach over 60 million tons in 2023,^[^ ^3^, ^4^ ^]^ and is associated with significant health^[^ ^5^ ^]^ and environmental^[^ ^6^ ^]^ impacts. RtR includes both a social movement, for example, the promotion of repair cafes and parties,^[^ ^7^ ^]^ and a drive for legislation to embed those principles in law.^[^ ^8^ ^]^ Some RtR laws now exist in the European Union,^[^ ^9^ ^]^ the United Kingdom,^[^ ^10^ ^]^ and in several U.S. state legislators.^[^ ^11^ ^]^

Repair organizations and collectives, including the US‐based “Repair Association”, founded in 2013,^[^ ^12^ ^]^ and European‐based “The Right to Repair Campaign”,^[^ ^13^ ^]^ aim to promote the repairable design of products so that they last longer. RtR is seen as a driving force to move away from the “take‐made‐dispose” model^[^ ^14^ ^]^ of production, toward a more circular mode of production and re‐use. Hernandez et al. identified five major constraints that restrict product repairability by consumers,^[^ ^15^ ^]^ thereby reducing the chance to move to a more circular economy. For the design of products that embrace RtR, these can also be seen *as principles to design against*. These five constraints are:

1. Lack of knowledge of how products work.
2. Lack of spare parts, technical information, and restricted contracts.
3. Lack of economic incentives to repair a product.
4. Lack of engagement, emotional and economic attachment to products.
5. Lack of design and manufacturing features promoting repairability.

Furthermore, the UN's 2022 Report on Sustainable Development Goals (UNSDGs)^[^ ^16^ ^]^ shows that the average worldwide E‐waste collection rate (where E‐waste is collected and managed in an environmentally sound way) is 22.8%, and this low rate is explicitly linked to the 12^th^ UNSDG (“Ensure sustainable consumption and production patterns”). Universities worldwide are seen as having a crucial role in creating a more sustainable future, as expressed through the UNSDGs.^[^ ^17^ ^]^ Universities can act as pillars, nationally, internationally, and locally, in driving sustainable development.^[^ ^18^ ^]^ Accordingly, in this paper, we outline a pilot study for teaching the principles of RtR to first‐year undergraduate students on a design‐engineering course. We intend this research to act as guidance for how RtR principles can be taught to undergraduate students from diverse backgrounds.

### 1.2 Sustainable Engineering Design in Education, Current Practices

The ability of graduate engineers to consider their work's ethical and sustainable impact has often been described as lacking. As outlined, Universities can play a key role in creating a sustainable future. Engineering education courses have begun to shift focus to address this, often with courses creating a greater focus on sustainability. Engineering learning outcomes for UK‐accredited university courses are defined by eighteen learning outcomes, through the Accreditation of Higher Education Programs (AHEP 4).^[^ ^19^ ^]^ Of these eighteen learning outcomes, two have links to sustainability:

- C5: Design solutions for complex problems that meet a combination of societal, user, business, and customer needs as appropriate. This will involve consideration of applicable health and safety, diversity, inclusion, cultural, societal, environmental, and commercial matters, codes of practice, and industry standards.
- C7: Evaluate the environmental and societal impact of solutions to complex problems and minimize adverse impacts.

Similar learning outcomes for higher education (HE) are demonstrated in other countries,^[^ ^20^ ^]^ with guidelines from ABET^[^ ^21^ ^]^ driving the requirements for sustainability in USA engineering degree programs. Although many universities follow the same learning outcomes, the approaches taken to achieve them are diverse. Some programs elect to run dedicated sustainable design modules within the course, for example, modules in Sustainability assessment for design,^[^ ^22^ ^]^ while some integrate sustainability within sections of the course such as UNSDGs in design modules.^[^ ^23^ ^]^ This can often lead to sustainability being taught in an abstract manner with difficulties in matching student perception of sustainability with the learning outcomes from the programs.^[^ ^24^ ^]^ In response to this Malz et al. developed a course for Bachelor's and Master's students in mechanical, biomedical, and industrial engineering, introducing a sustainable way of thinking, and training skills required to develop more sustainable solutions.^[^ ^25^ ^]^ This interdisciplinary approach aimed to promote the development of design competencies and social competencies within the project. The course covered material selection and material life cycle using GRANTA EduPack and sustainable design guidelines. This approach, combined with industry engagement, allowed students to learn and apply relevant basic methods from the field of product development and material selection. Other institutions have dedicated programs focusing on sustainable engineering at the Bachelor's^[^ ^26^ ^]^ and Master's levels.^[^ ^27^ ^]^ These aim to give a coherent and consistent thread of sustainability throughout the student's education, leading to increased awareness and an increased ability to solve problems using sustainable solutions.

Sánchez‐Carracedo et al. developed tools to embed and assess education for sustainable (ESD) development within engineering curricula.^[^ ^24^ ^]^ This states that for sustainability competencies to reach all students the curricula must include a significant number of compulsory courses in which they are developed.

Further frameworks for embedding sustainability within engineering education HE courses have been outlined by “Engineering for One Planet”^[^ ^28^ ^]^ with guidance to define what it means to be an engineer who is equipped to protect and improve our planet and lives. This work has been conducted in alignment with ABET accreditation standards. Implementation of this framework was conducted in a project‐based learning (PBL) course at Arizona State University,^[^ ^29^ ^]^ with students able to use and engage in a multi‐faceted way with each competency, making connections between competencies during their course.

Over the past decades, as ESD has gained traction in HE, there has been a corresponding increase in the literature reviewing it. Thus, some areas of deficiency have been identified along with suggestions by various authors. Zhang et al. outlined some of these challenges as^[^ ^30^ ^]^:

- Shifting paradigms around sustainability
- Rigidity of the existing education system
- Lack of new methods of teaching
- Lack of resources to teach sustainability

Thus, depending on the institution and place, the students will not be equally educated for future challenges including sustainability.^[^ ^31^ ^]^

### 1.3 Effective Methods of Teaching Sustainable Engineering Design

Comprehensive publications on incorporating ESD into engineering study plans are lacking.^[^ ^32^, ^33^ ^]^ Leiva‐Brondo et al. assessed university students' sustainability and UNSDGs knowledge, noting intermediate knowledge but low SDG awareness, highlighting the role of educational institutes.^[^ ^34^ ^]^ In the UAE, Al‐Naqbi et al. found strong positive attitudes but moderate behaviors toward ESD.^[^ ^35^ ^]^ A study at California Polytechnic State University revealed barriers to ESD adoption, including time constraints, lack of course promotion, and relevance issues.^[^ ^36^ ^]^ They suggested integrating sustainability into existing courses rather than creating standalone ones. Fisher et al. found that exposing students to sustainability in a class is more effective than multiple sustainability courses, with course type being more impactful than quantity.^[^ ^37^ ^]^

Future engineers must acquire a broad skill set, encompassing both technical and non‐technical abilities. Vital soft skills such as independent learning, critical thinking, handling criticism, and social interactions are best developed through active student engagement in the learning process.^[^ ^38^ ^]^ Lozano et al. compared a variety of pedagogical approaches in their effectiveness in developing sustainability competences.^[^ ^39^ ^]^ They observed that even though lecturing and case studies are among the most widely used techniques, they were the least effective in ESD. Active approaches like project or problem‐based learning, while working in interdisciplinary teams, were found to be the most effective. Segalas et al. also mentioned that courses with community‐oriented and constructive pedagogies were highly effective in developing students' knowledge of sustainable development.^[^ ^40^ ^]^ Howell presented the findings from an interdisciplinary ESD course that used the “flipped classroom” design where students prepared for the core content before coming to the classroom, while the in‐class sessions had more active‐leaning exercises, reflective practices, and mini‐lectures. More than 90% of the students reported the classes to be more engaging when using this approach for ESD.^[^ ^41^ ^]^ Barth et al. considered the importance of both formal and informal learning for the development of ESD competencies. They especially talked about the role of interdisciplinarity and the need for students' self‐responsibility, where some of the learning happens without explicit assistance from the instructor, for example, the research or skill development outside the class.^[^ ^42^ ^]^

### 1.4 Research Aims and Questions

The overwhelming themes emerging from engineering and design education literature show evidence that project‐based University programs, can engage students and help to embed fundamental principles, practical and technical skills, and specific knowledge focused on UNSDGs. This highlighted that a PBL‐based course, teaching the principles for sustainable design, toward tackling UNSDG 12, could be highly effective. While the RtR is becoming an important societal movement, we have not yet noted many examples of tackling this problem explicitly in engineering PBL courses. Some of the prescribed learning outcomes in UK and international engineering degrees align well with RtR principles, and consequently, it is a relevant topic.

Accordingly, in this paper, we present and analyze a novel pilot study, for a first‐year PBL university engineering design course, where students are given a project to design and make a household product according to RtR principles. We initially present an overview of the course design, including the content, assessment, and pedagogy. To evaluate the impact of the project on students, we used an anonymous questionnaire and conducted a focus group, which we used alongside their outputs to give an overview of what student priorities are, and what they learned during the module. All questions asked and the data are included in Supporting Information. Our aims and evaluations in presenting this work focus on:

- Detail the design of a multi‐disciplinary engineering design module that teaches engineering science fundamentals, design and ideation, analysis skills, hands‐on skills, and teamwork.
- A resource to show engineering educators some of the possible benefits of teaching in a project‐based way, even in the initial stages of an engineering degree.
- Documenting the successful implementation of RtR principles, employing engineering science to achieve technically viable solutions, and measuring student engagement through the module.
- Assessing the motivation of students and progress toward becoming future engineers who are engaged in solving global challenges.

We finish off this paper by presenting some lessons and takeaway points for educators.

## 2 Context and Overview of the Prototyping Module

### 2.1 Education Setting and Prior Learning

The module (or “class”) we discuss in this paper is called “Prototyping”. It is in the first year of the “Global Design Engineering” degree which is a design‐focused general engineering degree at The Engineering and Design Institute (TEDI‐London), a specialized HE provider in the United Kingdom which teaches almost entirely using PBL. Each year in TEDI‐London comprises three, 10 weeks long, teaching blocks, and Prototyping took place in the second block of the year. In the prior block, students had two concurrent modules: “Introduction to Engineering Design” (ITD) and “Reverse Engineering” (RE).

In the ITD module, students took part in the “Engineering for People, Design Challenge”,^[^ ^43^ ^]^ which is an international competition set by “Engineers without Borders”. Students were tasked to define a problem and design a solution, taking into account social, environmental, and economic considerations. They mainly focused on developing a concept rather than a physical prototype.

In the RE module, students were tasked to tear down a consumer product (a robot vacuum cleaner, RVC), investigate its function, and mathematically analyze the behavior of internal components (motors and batteries, for example). RE provided students with experience in investigating many of the core components of RVC and how they function and fit together. This was useful for the design and build phase of the upcoming Prototyping module.

### 2.2 Content‐assessment‐pedagogy Framework for the Module Design

**Figure 1:**
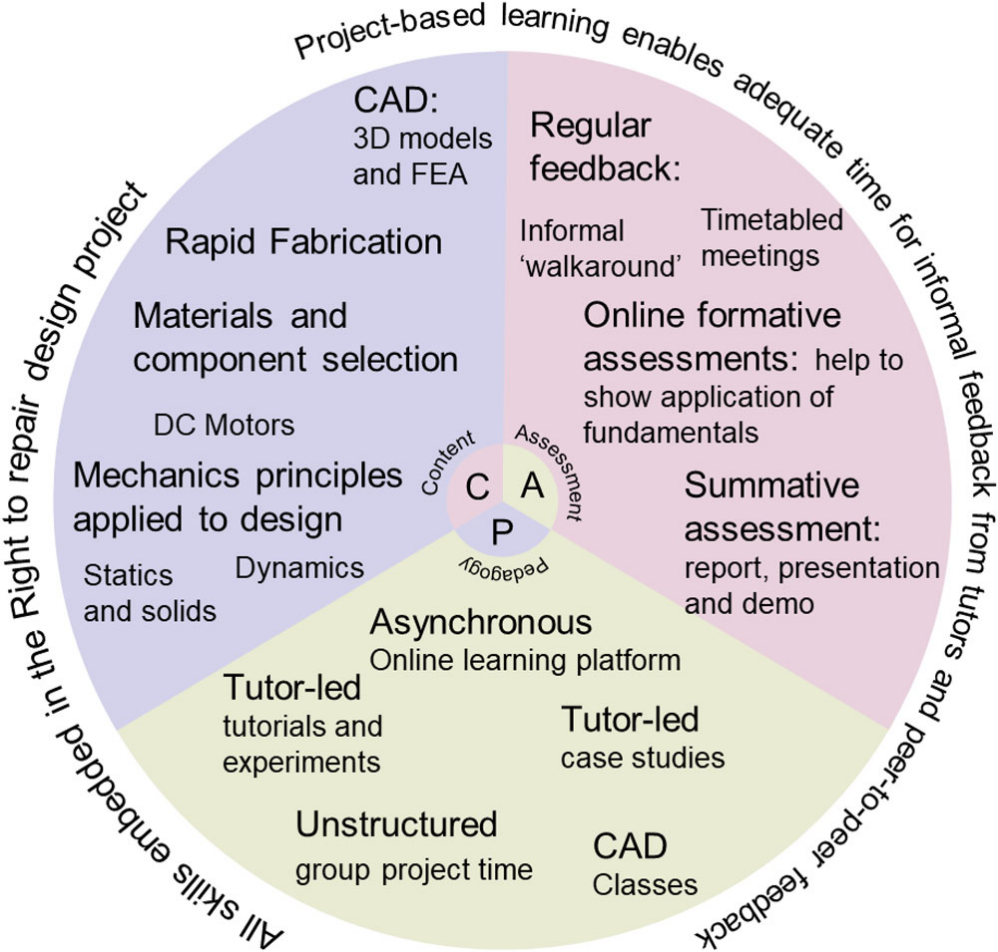
Infographic showing the overview of the content, assessment, and pedagogy approach to the design of the “Prototyping” module.

The initial design of the Prototyping module was constructed using the content‐assessment‐pedagogy (CAP) framework.^[^ ^44^ ^]^ This explicitly links the goal and specification of what we want the students to learn (content), how we measure the student's progress against that specification (assessment), and how we implement that (pedagogy). We summarize the course construction in **Figure** **1**, which links together the three CAP components. Team‐based PBL enables a broad range of learning opportunities,^[^ ^45^ ^]^ some of which can be classed as the “enduring outcomes”,^[^ ^44^ ^]^ which are the most fundamental outcomes that occur during the learning experience. The enduring outcomes are complemented by the “important‐to‐know” outcomes that are needed to achieve the enduring outcomes. Other learning opportunities are classed as “good‐to‐be‐familiar‐with” outcomes, which benefit learners but are not crucial to the learning experience.

The enduring outcomes are to embed the skills required for prototyping and demonstrate these as a key step for evaluation and problem‐solving within the user‐centered design process, while also developing students' understanding of sustainable design more broadly. Some other “enduring outcomes” include building students' ability to sketch, develop and iterate ideas with colleagues, and produce CAD models. Students must also develop and apply basic engineering models to inform a design, for example, to calculate the required torque from a DC motor, which will give a suitable kinematic performance of the RVC. Furthermore, students should engage in developing and coding, using Arduino, a relevant algorithm for the RVC, for example, a system to detect walls and change the motor direction as a result. Not every student in each team necessarily engages in physically prototyping the Arduino, hence, this is considered important‐to‐know outcomes. Other important‐to‐know outcomes include reading and analyzing technical literature, implementing design for additive^[^ ^46^ ^]^ and subtractive manufacturing principles, and justifying material selection. Good‐to‐be‐familiar‐with outcomes include project planning and management, overcoming group work problems, and clarity of report writing and presentation.

### 2.3 Design of the Prototyping Module

In the Prototyping module, students were tasked to redesign and build the RVC. Students had to consider the environmental impact of their material and design choices; focusing on how these affect the lifecycle of the product under the RtR movement. The module was split into three phases; 1) ideation and planning, 2) building and implementation, and 3) test and evaluation. Cycling back through these phases was encouraged as part of the user‐centered design process.

The content was presented in a blended fashion, with online material to cover fundamental topics including, mechanics, electrical science, manufacturing, CAE, and design principles; and in‐person sessions twice a week for 4 h each. These sessions were split between tutorials, case studies, and unstructured group project time, which comprised the majority of the time. The size of the teams was 3 to 5 students. Students were assessed through a group presentation (including a demonstration and submission of the physical prototype of their RVC) and an individual report, with high marks awarded for exceptional practical skills, improving through iteration, and insightful engineering analysis of the components.

**Figure 2:**
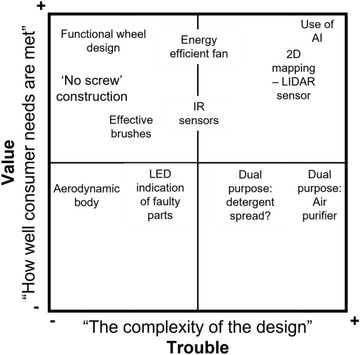
Example of representative student work (rewritten for clarity by the authors), considering the impact of different initial design features on a 2 × 2 matrix, trouble of implementation plotted against the added value to the user.

During the ideation and planning phase, students were presented with data highlighting the magnitude of the E‐Waste problem, and then with a series of case studies^[^ ^47^ ^]^ on how others had designed repairable electrical products. Some of the principles discussed by Hernandez et al., including creating an emotional attachment to products, economic incentives, modularity, and easy disassembly^[^ ^15^ ^]^ through manufacturing innovation were evident in the case studies. After this, students were invited to ideate for implementing RtR principles in their own designs. Students used a 2 × 2 matrix (an example is shown in **Figure** **2**) to rapidly identify opportunity areas and prioritize^[^ ^48^ ^]^ their work in an effective way. Following this, students were invited to sketch and list their ideas, apportion work, and create a work plan, with most groups creating a Gantt chart. During this planning stage, students were provided technical training on coding (including using the TinkerCAD Arduino emulator) and using hardware Arduino boards, as these skills were required for RVC control.

In the building and implementation phase, students were encouraged to start designing and fabricating their prototypes. Students were not prescribed set tasks during this period and were encouraged to plan and make their own decisions. Most of the time was spent in group project time where they could decide on how best to progress the project. Aside from such unstructured time, students had tutor‐led CAD sessions where they were given tasks to help prepare them for designing the prototypes. They were also given free time where they could ask for guidance on how to implement their design in CAD. Students were also given some tutorial sessions covering mechanics fundamentals to help them design their prototypes. For example, they underwent a case study of a remote‐controlled car, where they calculated the required angular velocity and torque for a given speed and acceleration. Makerspace technicians were available to provide hands‐on support for fabrication, such as 3D printing, laser‐cutting, or microcontroller implementation.

In the testing and evaluation phase, students were required to evaluate, improve, and iterate their designs. The types of evaluations were varied and could include:

- RtR scope: how simple would it be to repair, should a certain component fail?
- The electromechanical design: is the RVC speed appropriate for the task? How could this be altered? Does the RVC appropriately dodge obstacles?

Students were encouraged to carry out testing and evaluation throughout their building and implementation and circle back between these phases. During this final phase, students completed an individual report and a group presentation for their assessment.

## 3 Results and Findings

The prototyping module was carried out over ten weeks of teaching time. To gather information a survey and focus group was conducted. Out of 40 registered students, 14 participated in the survey and 5 were part of the focus group. The following subsections present the findings for the two research questions posed in Section 1.

### 3.1 Successful Implementation of RtR Principles, Engineering Science Outcomes, and High Engagement

We planned to measure a successful implementation of RtR principles by gathering evidence, in the student work, for the five design constraints by Hernandez et al. and the two AHEP learning outcomes (as mentioned in Section 1).

**Figure 3:**
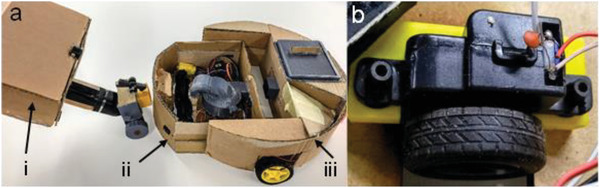
a) This modular RVC prototype includes three sections, i: handle for upright usage, ii: suction and cleaning parts, iii: RVC propulsion and motors. b) Shows the removable rubber feet to connect the DC motor and wheel.

**Figure** **3**a shows a student example of an RVC design. This modular design has three detachable components: section i, a detachable handle, section ii, suction components and section iii, propulsion unit (powered by 2 DC motors). Combining a detachable handle with suction components, one obtains a traditional stand‐up vacuum cleaner, and when suction components are used with a propulsion unit, it works as an autonomous RVC. The design was an innovative response to design constraint 5: “ Lack of design and manufacturing features promoting repairability”. This group also demonstrated thorough mathematical modeling and specification of the electromechanical components in the RVC, showing excellent performance in relevant AHEP learning outcomes, including C2: the analysis of complex problems using mathematics and engineering science principles, and C3: applying analytical techniques to model complex problems.^[^ ^19^ ^]^

Figure 3b shows another innovative student design covering design constraint 5. Students in this group researched that motors may often need to be replaced, as they were low‐cost brushed DC motors, and may often become clogged with hair. They designed rubber connectors to hold the motor housing and wheel in place. This secured the motor into place but ensured that the component could be easily removed and replaced by hand, without opening the whole RVC. This also implemented design constraint 2 (Lack of spare parts, technical information, and restricted contracts) because the design allowed for a generic motor that could be replaced by any similar model. One student, recalling this design during the focus group, showed a creative source of inspiration for this:

Student C: “My group decided to put rubber footings inside the casing, so it would allow for people to easily remove different components, this was a really great way to not have screws. This is more sustainable, as you wouldn't have to throw it away (if broken). Our initial thought was what you use for your coat, the poppers. When you close it, it won't move, but then you can easily remove it.”

The freedom to design, and the relatively unpressured environment where innovation was encouraged, proved to be major aspects of the module which drove engagement. For example, when asked (in the anonymous survey) “What were your favorite parts of the project during Prototyping? What interested you the most?” some representative responses included:

- *“The creative freedom and challenging prompts.”*
- *“The first part of bringing your own ideas and imagining your own robot vacuum. Then to try implement these ideas to create a more sustainable one while following the brief.”*
- *“One of the most interesting things is the fact that we can use our creative freedom and prototype our own iterations.”*

**Figure 4:**
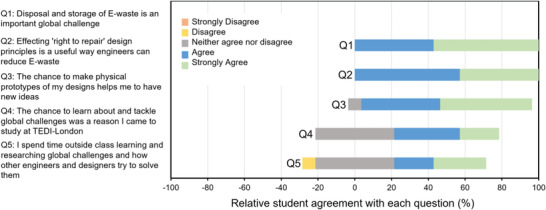
Summary of data from a short, anonymous questionnaire on students' motivations around designing to help tackle global challenges in their degree.

In the survey responses, as shown in **Figure** **4**, most students found motivation in the chance to learn about and tackle global challenges in their degree with 100% agreeing or strongly agreeing with Q1 and Q2 of the survey. A further 57.1% agreed (in responses to Q4) that the chance to tackle such problems was a specific motivation in choosing the course at TEDI‐London.

**Figure 5:**
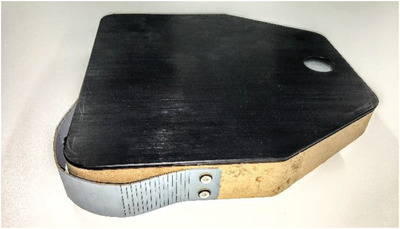
Example of student work that demonstrated high aesthetic design and quality, aimed to help foster an emotional engagement with the product.

Another of the RtR design constraint was 4: “Lack of engagement, emotional and economic attachment to products”. One of the groups took this challenge to heart by creating a prototype with a strong visual style, as shown in **Figure** **5**. This group produced several iterations of the prototype, with a high level of visual flair shown in the final prototype, with inspiration taken from a sleek weighing scale. The design differences between examples shown in Figures 3 and 5, respectively, highlighted the freedom students had in pursuing their own ideas, artistic vision, and innovations, which echoes the clear findings from Wang who showed that the chance to implement individual choice and design flair in a project is a significant driver of engagement and motivation.^[^ ^49^ ^]^

### 3.2 Motivation of Students and Progress toward Becoming Future Engineers Who Are Engaged in Solving Global Challenges

**Figure 6:**
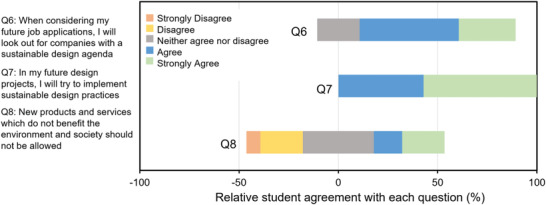
Summary of data from a short, anonymous questionnaire on considerations of applying their knowledge to future projects and engagement for the future career.

We noted many examples of innovative design practice we observed through the practical, prototyping phase of the project. Furthermore, in feedback from the focus group, we found evidence that students have embedded the sustainable design principles taught through the module and had started to evidence them in their wider work. The survey results, as summarized in **Figure** **6**, showed that companies having a sustainable design agenda were a consideration for 78.6% of respondents. Furthermore, the results show that 100% of respondents agree or strongly agree that they will try to implement sustainable design practices in future projects. A quote from the focus group helped to highlight the depth of feeling students hold for sustainable design:

Student E: “It's all kind of instilled in us now. I don't think… after those projects… it's possible to conduct any project or go into anything without thinking about these things [sustainable design practices]. That's just how we've been trained.”

Another student added the following quote below, which, in summary, demonstrates the belief that, in order to reduce E‐waste, in some cases it is best to not design a product at all.

Student A: “I feel like one of the things that kind of just like, is it necessary? There are things we were [considering] designing, wanting to make, but we now think: is this actually needed? It's just like a waste of the resources we have, it might be nice, but is it necessary in the first place? It's made me pretty angry at some stuff that I wasn't beforehand… look how many custom parts there are here… you can't buy any of these anymore. Why have they done this? Why is this all proprietary? It's really frustrating, so much stuff, but it's like they didn't think in the slightest about what we were learning in Prototyping,”

He and Gu presented a methodology for sustainable design, to aid in the generation of optimized products with a low environmental impact,^[^ ^50^ ^]^ thereby avoiding the creation of products with poor environmental impact. They present a more systematic method for our students' desire to avoid designing unsustainable products, and this type of framework may be fruitful for a further, more specialized study. It is interesting to note that the response to Q8 in the survey (“New products and services which do not benefit the environment and society should not be allowed”) had only 35.7% of agreement from respondents. This suggests that our cohort may hold more moderate views on sustainability policies.

### 3.3 Limitations to the Study

TEDI‐London is a new, specialist HE institute that presently teaches one degree program, a bachelor's degree in “Global Design Engineering”. Thus, the students here do not completely represent the wider engineering cohort in the UK. In the country, the distribution of engineering degree subjects taken in 2020/2021 was^[^ ^51^ ^]^: Mechanical (22.5% of students), Electrical and electronic (15.8%), General (15.7%), and Civil (13.7%), showing that most engineering undergraduates take specialized single‐discipline degrees. Global Design Engineering has a broad scope similar to General Engineering.

Due to the current small size of TEDI‐London, this pilot study has a small sample size without a separate control group. However, the insights and sustainability‐themed course design principles tested and presented here are significant. They will pave the way for broader research studies in the future.

## 4 Conclusion

Overall, the teaching team found the Prototyping module to be a satisfying one to teach, with strong engagement and attendance from the large majority of students. There was quantifiable evidence to suggest that the enduring outcomes of the module, including the technical skills required for prototyping, development, and ideation were well practiced through the module, and a large majority of students showed a marked improvement in these abilities. These enduring outcomes were evidenced by the quality of the physical prototypes produced, and the reflections from the questionnaire, focus group, and the formal reports. Students' ability to apply engineering science models to inform a design was mixed, with some students demonstrating excellent analytical skills (for example, torque calculations, dynamics, and stress analysis), while others went into less depth. As a teaching team, we reflect that we may need to include better guidance on how to achieve excellence in this area through more explicit marking guidelines.

Of the five design principles^[^ ^15^ ^]^ for RtR espoused by Hernandez et al., students clearly designed around three of them, with some innovative examples of problem‐solving and design. The remaining two were: “lack of knowledge of how products work” and “lack of economic incentives to repair a product”. While these principles are valid, the specific learning outcomes of this module did not encompass these themes, and thus the students avoided doing work that would not gain them credit in the formal assessments. We believe that students in different types of degree programs such as, for example, Engineering Management may be better suited to tackle these types of problems, particularly if they were part of a multidisciplinary team.

### 4.1 Key Takeaways for Educators

**Figure 7:**
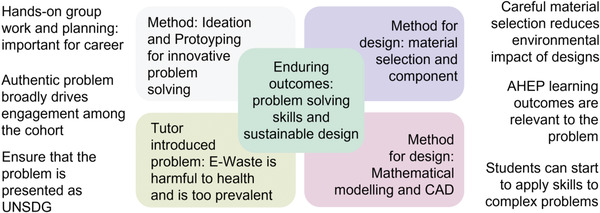
Diagram highlighting some of the key themes of creating an engaging PBL course.

The learning environment at TEDI‐London, which primarily employs PBL, differs from traditional engineering HE institutions; however, it is important to note a growing trend toward the adoption of problem‐based and PBL methodologies,^[^ ^52^ ^]^ This trend enables institutions to learn from one another and implement lessons derived from PBL experiences. Accordingly, here we have set out some summarized points to help give clear points for educators to take away. Furthermore, we have summarized some of our findings in the diagram in **Figure** **7**.

The summary points are:

1. Embedding a project with sustainable themes, which is directly linked to some of the UNSDG, strongly drives engagement for a wide range of students.
2. Knowledge appears to be well embedded by the end of the module, and students are keen to apply their skills to a range of future projects. They showed deeper engagement with the “designed world”. They recognize when products are designed poorly and are especially unhappy with the “planned obsolescence” approach to design.
3. Where applicable, physical making and rapid prototyping helps to drive innovation and engagement.
4. Students were highly motivated to show off their findings to others; in some cases, leading to further excitement and engagement.
5. Most students wish to work for companies that have a sustainable agenda. They believe that they themselves can help to drive change if they have the correct training.
6. Projects that involve relevant technical analysis increase the motivation of engineering students. For example, in this case, students could specify DC motors, carry out kinematic analysis, employ CAD for casing, and do tensile testing of materials. The choice of a small‐scale device, with many small components and sensors provided a range of technical tasks for students in this project.
7. Groups should have students with a range of skill levels, and progress needs to be monitored regularly to ensure that all students feel that their teammates are pulling their weight.
8. Structuring PBL with significant tutor‐led activities at the start of the module, and then increasingly allowing students to guide their own learning worked well in this module.

## Conflict of Interest

The authors declare no conflict of interest.

## Supporting information

Supporting Information

## Data Availability

The data that support the findings of this study are available from the corresponding author upon reasonable request.
