# Supplementary material for: Sustainable Engineering Design in Education: A Pilot Study of Teaching Right‐to‐Repair Principles through Project‐Based Learning
Source: Glob Chall. 2023 Oct 3;7(10):2300158. doi: 10.1002/gch2.202300158 (PMC10566800; doi:10.1002/gch2.202300158)
Supplement: Supplementary file 1 — Supporting Information [file GCH2-7-2300158-s001.pdf]

Global Challenges

Open Access

Supporting Information

for *Global Challenges.*, DOI 10.1002/gch2.202300158

Sustainable Engineering Design in Education: A Pilot Study of Teaching Right-to-Repair Principles through Project-Based Learning

Sam Fishlock, Matthew Thompson and Anoop Grewal*

Supporting Information

Sustainable engineering design in education: a pilot study of teaching right-to repair principles through project-based learning

*Sam Fishlock *, Matthew Thompson , Anoop Grewal.*

Full list of questions asked in the anonymous questionnaire sent to all students.

1. Think back to 'ENG 1201 Prototyping'. What were your favourite parts of the project during Prototyping? What interested you the most?
2. Think back to 'ENG 1201 Prototyping'. What design practice were you most proud of?
3. Think back to 'ENG 1201 Prototyping'. What aspects of your project and design related to sustainability?

Rate how much you agree with each of the following statements.
'Global challenges' relates to the major problems faced by humanity and our planet. Some Global Challenges include reducing poverty and hunger, the design of sustainable cities and communities, and creating affordable and clean sources of energy.

	Strongly Disagree	Disagree	Neither agree nor disagree	Agree	Strongly Agree
Disposal and storage of E-waste is an important global challenge				42.9 %	57.1 %
Effecting 'right to repair' design principles is a useful way engineers can reduce E-waste				57.1 %	42.9 %
The chance to learn about and tackle global challenges, (for example			42.9 %	35.7 %	21.4 %

reducing E-waste), was a reason I came to study at TEDI-London					
I spend time outside class learning and researching global challenges and how other engineers and designers try to solve them		7.1 %	42.9 %	21.4 %	28.6 %
The chance to make physical prototypes of my designs helps me to have new ideas			7.1 %	42.9	50 %

Table S1. Full results of questions 1 to 5, asked of students who completed ‘Prototyping’.

	Strongly Disagree	Disagree	Neither agree nor disagree	Agree	Strongly Agree
When considering my future job applications, I will look out for companies with a sustainable design agenda			21.4 %	50 %	28.6 %
In my future design projects, I will try to implement sustainable design practices				42.9 %	57.1 %
New products and services which do not benefit the environment and society should not be allowed	7.1 %	21.4 %	35.7 %	14.3 %	21.4 %

Table S2. Full results of questions 1 to 5, asked of students who completed ‘Prototyping’.
